# In Vitro Three-Dimensional Human Liver Model for Drug-Induced Liver and Bile Duct Injury Prediction

**DOI:** 10.3390/toxics14080724

**Published:** 2026-08-14

**Authors:** Xiaonan Fu, Jiangping Hu, Xintong Jiang, Yedan Sun, Wanling Xiang, Rong Kuang, Hua Kang, Licheng He, Jing Sang

**Affiliations:** 1 Zhejiang Institute for Food and Drug Control, Hangzhou 310052, China; fuxiaonan@zjyj.org.cn (X.F.); sunyedan@zjyj.org.cn (Y.S.); xiangwanling@zjyj.org.cn (W.X.); kuangrong@zjyj.org.cn (R.K.); kuanghua@zjyj.org.cn (H.K.); helicheng@zjyj.org.cn (L.H.); 2 Zhejiang Key Laboratory of Biopharmaceutical Contact Materials, Hangzhou 310052, China; 3 NMPA Key Laboratory for Animal Alternative Testing Technology of Cosmetics, Hangzhou 310052, China; 4 School of Pharmacy, China Pharmaceutical University, Nanjing 210009, China; hjp18457505306@163.com; 5 Department of Pharmacy, China Jiliang University, Hangzhou 310018, China; 15803054430@163.com

**Keywords:** in vitro liver model, three-dimensional culture, drug-induced liver injury, DILI prediction

## Abstract

In drug-induced liver injury (DILI) prediction field, animal models and in vitro cell models are most commonly used. However, animal models require long experimental timelines and may exhibit species-specific differences compared with humans, whereas conventional two-dimensional (2D) cell culture models lack cell-to-cell and cell-to-extracellular matrix (ECM) interaction. Liver organoid models and liver organ-on-a-chip can better simulate the human liver microenvironment; however, the construction of liver organoids requires a long cycle and high costs, while liver organ-on-a-chip systems demand specialized equipment and professional technicians. Herein, we selected the human C3A cell line, characterized by its low cost and facile culture conditions to establish an in vitro three-dimensional (3D) liver model. Briefly, C3A cells were embedded in Matrigel and cultured for 7 days to allow model maturation. Compared with their 2D-cultured cell model, the established 3D model exhibited elevated mRNA expression levels of drug-metabolizing cytochrome P450 enzymes (CYPs). Moreover, the model displayed robust expression of key hepatic biomarkers, as well as bile duct biomarkers. To evaluate the model’s applicability for DILI prediction, we performed toxicity assessments using a panel of six well-characterized hepatotoxicants and three non-hepatotoxic compounds. Notably, the 3D C3A model achieved a sensitivity of 83.3%, specificity of 100%, and overall accuracy of 88.9%. Furthermore, treatment of this model with chlorpromazine, a well-characterized cholangiotoxic agent, resulted in suppressed expression of the bile duct biomarker cytokeratin 19 (CK19) and bile salt export pump (BSEP), accompanied by impaired bile acid transport capacity. Taken together, this study provided a simple, low-cost, easy to culture, and more readily scalable 3D hepatic model in comparison with conventional 2D primary human hepatocyte (PHHs) models and other advanced 3D liver models. Notably, the model displayed dual hepatic and biliary characteristics, supporting predictions of both DILI and drug-induced bile duct injury. It provided a promising in vitro platform for assessing drug-induced hepatobiliary toxicity, with potential to reduce reliance on animal experiments and accelerate early-stage screening of novel pharmaceutical candidates.

## 1. Introduction

Liver is the core organ for drug metabolism and also a primary target organ for adverse drug reactions. DILI is the leading cause of drug failure during preclinical or clinical trial phases, and one of the main reasons for drug withdrawal and clinical usage restrictions [1,2]. Through preclinical DILI prediction, candidate drugs with hepatotoxicity can be screened out, avoiding research termination due to hepatotoxicity issues in later stages, significantly shortening the development cycle, reducing economic costs, and ensuring patient health.

In the traditional DILI prediction field, animal models and 2D cell culture models are most commonly used. Animal models and 2D hepatocyte cultures have constituted the backbone of regulatory toxicology for over six decades, delivering contributions that remain foundational to contemporary drug development. However, animal experiments have long cycles and high costs. Moreover, due to species differences between animals and humans, animal models may fail to accurately predict DILI in humans. As an illustration, preclinical safety assessments covering 150 pharmaceutical compounds reported predictive accuracies of only 63% and 43% in non-rodent and rodent animals, respectively, with the lowest accuracy observed in the hepatobiliary system [3]. Additionally, considering animal ethics and welfare, institutions advocate the use of alternative methods for drug toxicology research [4,5]. Conventional 2D cell culture models have advantages such as simple operation, easy cultivation, short cycle, and suitability for high-throughput screening. Human tissue-derived primary cells, in particular, have shown potential in the DILI prediction field. For example, PHHs are considered the gold standard among 2D cell models for in vitro DILI prediction [6]. However, cells cultured in monolayer lack cell–cell and cell-extracellular matrix interactions differ significantly from human cells in spatial structure, which prevents precise simulation of the human physiological environment and consequently affects DILI prediction [4]. For instance, predicting DILI using 2D PHHs with simple cytotoxicity endpoints yielded an accuracy of only 67%, indicatong that the data obtained from conventional 2D cultures cannot be fully extrapolated to in vivo conditions [5]. Based on the above considerations, an in vitro model constructed from human cells that can better simulate the human liver has become an urgent need in the DILI prediction field.

In recent years, New Approach Methodologies (NAMs) have advanced rapidly and gained recognition from regulatory authorities worldwide. For instance, liver organoid models and liver organ-on-a-chip can better simulate human liver microenvironment, hold great potential for applications in DILI prediction and pharmaceutical evaluation of candidate drugs [7]. However, the construction of liver organoids requires a long cycle and high costs, while liver organ-on-a-chip systems demand specialized equipment and professional technicians. 3D cell culture technology can enhance cell–cell and cell-extracellular matrix interactions, enabling in vitro cells to be structurally and functionally closer to in vivo cells; therefore, the 3D in vitro liver models have promising prospects in DILI prediction. Typically, cells can be seeded in specially treated containers to form 3D cell models through cell differentiation or self-assembly. Alternatively, cells can be mixed with hydrogel and seeded in cell culture plates or printed into 3D cell models using 3D bioprinters [8].

Currently, the cell sources utilized to construct in vitro 3D hepatic models include PHHs, hepatocytes derived from induced pluripotent stem cells (iPSCs), HepaRG cells, and HepG2 cells [9]. PHHs exhibit high CYP enzyme expression levels and obvious drug responses, but they have limited sources, low expandability, and short lifespans [10]. iPSCs can be expanded for multiple generations, and the hepatocytes derived from iPSCs show moderate CYP enzyme expression and drug responses, but the induction of iPSCs into hepatocytes requires multiple cytokines and a long induction cycle, resulting in high economic and time costs [11]. HepaRG cells have a high expansion potential with moderate CYP enzyme expression and drug responses, but their culture requires multiple cytokines, and the induction of differentiation into mature hepatocytes takes a long cycle [12,13]. HepG2 cells are easily obtained, low-cost, immortalized, and readily expandable, but they have low CYP enzyme expression and weak drug responses [14]. C3A cells are a clonal derivative of HepG2 cells. Compared to HepG2, C3A cells exhibit stronger contact inhibition growth characteristics and can secrete more liver markers such as albumin (ALB) and alpha-fetoprotein (AFP). C3A cells have been widely applied in hepatotoxicity research [15,16].

This study selected C3A cells, which are human-derived, low-cost, and easy to culture, for the construction of an in vitro 3D liver model. C3A cells and Matrigel were mixed at a specific ratio and seeded in cell culture plates, allowing C3A cells to grow into a mature 3D liver model to enhance the biomimetic properties of the model. Notably, the model displayed dual hepatic and biliary characteristics, supporting predictions of both DILI and drug-induced bile duct injury.

## 2. Materials and Methods

### 2.1. Cell Culture

C3A cells were obtained from Xellar Biosystems. The culture medium was EMEM medium containing 10% fetal bovine serum and 1% penicillin–streptomycin. Cells were passaged upon reaching approximately 80% confluence. They were treated with 0.25% trypsin–EDTA solution for 2 min, after which the digestion was quenched with complete medium. Cells were passaged at least once before model construction, in this study, P10 C3A cells were used for the construction of 2D and 3D models. For monolayer cell culture, the cell seeding density was 1 × 10^4^ cells/cm^2^, which was experimentally optimized in this study.

### 2.2. 3D Cell Model Construction

The volume ratio of cell suspension to Matrigel (Corning, Corning, NY, USA, Cat. No. 354277, Lot No. 10124002) used for model construction was 1:2. The cell densities were selected based on preliminary optimization experiments, the density of the low-density 3D cell model was 300 cells/μL, and the density of the high-density 3D cell model was 3000 cells/μL. Typically, 6 μL of cell and Matrigel mixture was seeded per well in 96-well plates. After seeding, the culture plates were placed in a 37 °C, 5% CO_2_ and 95% humidity incubator and incubated in an upright position for 5 min, then inverted for another 5 min. After incubation, 100 μL of culture medium was added to each well, and the plates were returned to the incubator for culture. The model was considered mature when CYP3A4, CYP2C9, CYP2D6, and CYP1A2 mRNA expressions were significantly upregulated compared to 2D C3A cells at day 3 (*p* < 0.05) and when positive protein expressions of ALB, CYP3A4, CK19, and BSEP were detected.

### 2.3. RNA Extraction

Monolayer-cultured C3A cells or 3D C3A cell models were collected on designated days. Monolayer-cultured cells were washed three times with pre-cooled DPBS, and 0.5 mL of Trizol reagent was added per well (24-well plate) to lyse cells. The lysate was collected into 1.5 mL EP tubes. 3D C3A cell models were washed once with pre-cooled DPBS, and 1 mL of pre-cooled Cell Recovery Solution was added per well (24-well plate). The matrix was dissolved by thorough pipetting, and the solution was collected into 15 mL centrifuge tubes and centrifuged at 200× *g* for 5 min. The supernatant was discarded, complete removal of Matrigel was confirmed, and 0.5 mL of Trizol reagent was added to the cell pellet to lyse the cells. The lysate was collected into 1.5 mL EP tubes. To each tube of lysate, 0.1 mL of chloroform was added, vigorously shaken for 15 s, and left at room temperature for 2–3 min. The mixture was centrifuged at 12,000× *g* at 4 °C for 15 min. The upper aqueous phase was transferred to a new EP tube, and an equal volume of isopropanol was added, gently mixed by inversion, and left at 4 °C for 30 min. The mixture was centrifuged at 12,000× *g* at 4 °C for 10 min. The supernatant was discarded, and the RNA pellet was washed three times with 75% ethanol solution prepared with DEPC water. The RNA pellet was dissolved in DEPC water, and the RNA concentration was measured on a NanoPhotometer N60 (IMPLEN), with A260/A280 ratios ranging from 1.8 to 2.2 and A260/A230 ratios > 1.8, indicating high purity without protein or organic solvent contamination. All experiments were performed with three independent biological replicates and each sample was run in triplicate.

### 2.4. RT-qPCR

Reverse transcription was performed using the PrimeScript RT reagent Kit (Perfect Real Time) from Takara Bio Inc., Dalian, China, according to the manufacturer’s instructions. A 10 μL reaction system was used, containing 500 ng of total RNA. The reaction conditions were 37 °C for 15 min, 85 °C for 5 s, and storage at 4 °C. qPCR was performed using the TB Green Premix Ex Taq (Tli RNaseH Plus) kit according to the manufacturer’s instructions. A 20 μL reaction system was used. The reaction conditions were pre-denaturation at 95 °C for 30 s, PCR reaction at 95 °C for 5 s and 60 °C for 34 s for 40 cycles, followed by melting curve generation. qPCR was performed on an Applied Biosystems QuantStudio 5. Primers were synthesized by Sangon Biotech (Shanghai) Co., Ltd., Shanghai, China (Table 1). Data analysis was performed using the △△Ct method. GAPDH was selected as a reference gene based on previous literature reports [3,17,18]. Specific amplification was confirmed by melting-curve analysis. Relative mRNA expression was calculated by normalizing to the expression level of target genes in 2D-cultured C3A cells on day 3, which was set as 1.

### 2.5. Immunofluorescence

Monolayer-cultured C3A cells or 3D C3A cell models were fixed with 4% paraformaldehyde on designated days. Monolayer-cultured cells were fixed at room temperature for 15 min, and 3D C3A cell models were fixed for 1 h. After washing with DPBS, permeabilization was performed using 0.1% Triton X-100 solution. Monolayer-cultured cells were treated at room temperature for 10 min, and 3D C3A cell models were treated for 30 min. After washing with DPBS, blocking was performed using 10% BSA at room temperature for 1 h. The blocking solution was removed, and primary antibody solution was added to cover the samples and incubated overnight at 4 °C. Primary antibodies for Albumin (ab207327, 1:500 for Immunofluorescence), CYP3A4 (ab124921, 1:400 for Immunofluorescence), CK19 (ab7754, 1:200 for Immunofluorescence), and BSEP (ab255605, 1:200 for Immunofluorescence) were purchased from Abcam. Primary antibodies against Albumin, CYP3A4, CK19, and BSEP were supplied at stock concentrations of 0.584, 0.132, 1.00, and 0.705 mg/mL, respectively. After washing three times with DPBS, secondary antibody solution was added to cover the samples and incubated at room temperature for 2 h. Goat anti-rabbit IgG H&L (ab150077, 1:500 for Immunofluorescence) and goat anti-mouse IgG H&L (ab150116, 1:500 for Immunofluorescence) were purchased from Abcam. Goat anti-rabbit IgG H&L and goat anti-mouse IgG H&L were both supplied at stock concentrations of 2 mg/mL. After washing with DPBS, nuclear staining was performed using Hoechst 33,342 (Solarbio) at room temperature for 15 min. Images were captured using an inverted fluorescence microscope (LEICA DMi8).

### 2.6. Drug Hepatotoxicity Detection

Ten drugs were selected for the experiment and all Cmax data were obtained from published studies [19,20,21,22,23,24,25,26,27,28,29]. These included three non-hepatotoxic drugs: fexofenadine [19], mannitol [20], streptomycin [21], six hepatotoxic drugs: acetaminophen [22], troglitazone [23], omeprazole [24], chlorpromazine [25], rifampin [26], cyclosporin A [27], and one drug with ambiguous hepatotoxicity: sitagliptin [28,29]. All drugs were purchased from MCE (MedChemExpress), Monmouth Junction, NJ, USA. Five concentrations were selected for hepatotoxicity detection for each drug (Table 2). Monolayer-cultured cells or 3D models were seeded in 96-well plates. Medium was changed on days 2 and 5, and drug treatment was performed on day 7 post-seeding. ATP detection was performed on day 3 post-drug treatment using the CellTiter-Glo 3D Cell Viability Assay Kit and CellTiter-Lumi Luminescent Cell Viability Assay Kit. A total of 100 μL of ATP detection reagent was added to each well, incubated for 10 min for 2D cells and 1 h for 3D models, and detected on a SpectraMax iD3 microplate reader. Each experiment was performed with at least three replicates. Relative cell viability was calculated as the background-corrected luminescence intensity of treated groups divided by the background-corrected luminescence intensity of the untreated control group within each assay.

### 2.7. Drug Bile Duct Toxicity Detection

To verify whether the 3D C3A model could predict drug-induced bile duct toxicity, the model was treated with chlorpromazine, a prototypical BSEP inhibitor with bile-duct toxicity, given its established mechanism of action and clinical relevance to drug-induced cholestasis [30,31]. Changes in the expression of the bile duct marker CK19 and bile salt export pump BSEP were measured, and changes in bile acid transport capacity were detected. Drug treatment was performed on day 7 post-model seeding. The maximum non-hepatotoxic concentration of chlorpromazine was selected. Three days after drug treatment, immunofluorescence detection for CK19 and BSEP was performed using the same method as in Section 2.5., immunofluorescence. Simultaneously, a bile acid transport experiment was performed. The drug-containing medium was changed to medium containing 5 μmol/L Cholyl-Lys-Fluorescein (CLF, AAT Bioquest, Pleasanton, CA, USA), incubated for 4 h, and bile acid transport was observed using an inverted fluorescence microscope.

### 2.8. Data Processing and Analysis

GraphPad Prism 8 was used for drug half-maximal inhibitory concentration (IC50) analysis, non-linear regression (curve fit) analysis was performed under XY analyses using the Dose–Response–Inhibition model. The ratio of IC50 to the maximum plasma concentration in humans (Cmax) was used as the criterion for assessing hepatotoxicity. Drugs were classified as hepatotoxic when IC50/Cmax ≤ 50, and as non-hepatotoxic when IC50/Cmax > 50 [32].

### 2.9. Statistical Analysis

Statistical analyses were carried out using GraphPad Prism 8. Statistical comparisons were performed separately at each time point. Normality was assessed for each group at every time point. One-way ANOVA followed by Tukey’s post hoc multiple comparisons test was applied for data satisfying normality assumption. For data that failed normality test, Kruskal–Wallis test, followed by Dunn’s post hoc multiple comparisons test, was used. Differences with *p* < 0.05 were considered statistically significant.

## 3. Results

### 3.1. Cell Model Growth

**Figure 1:**
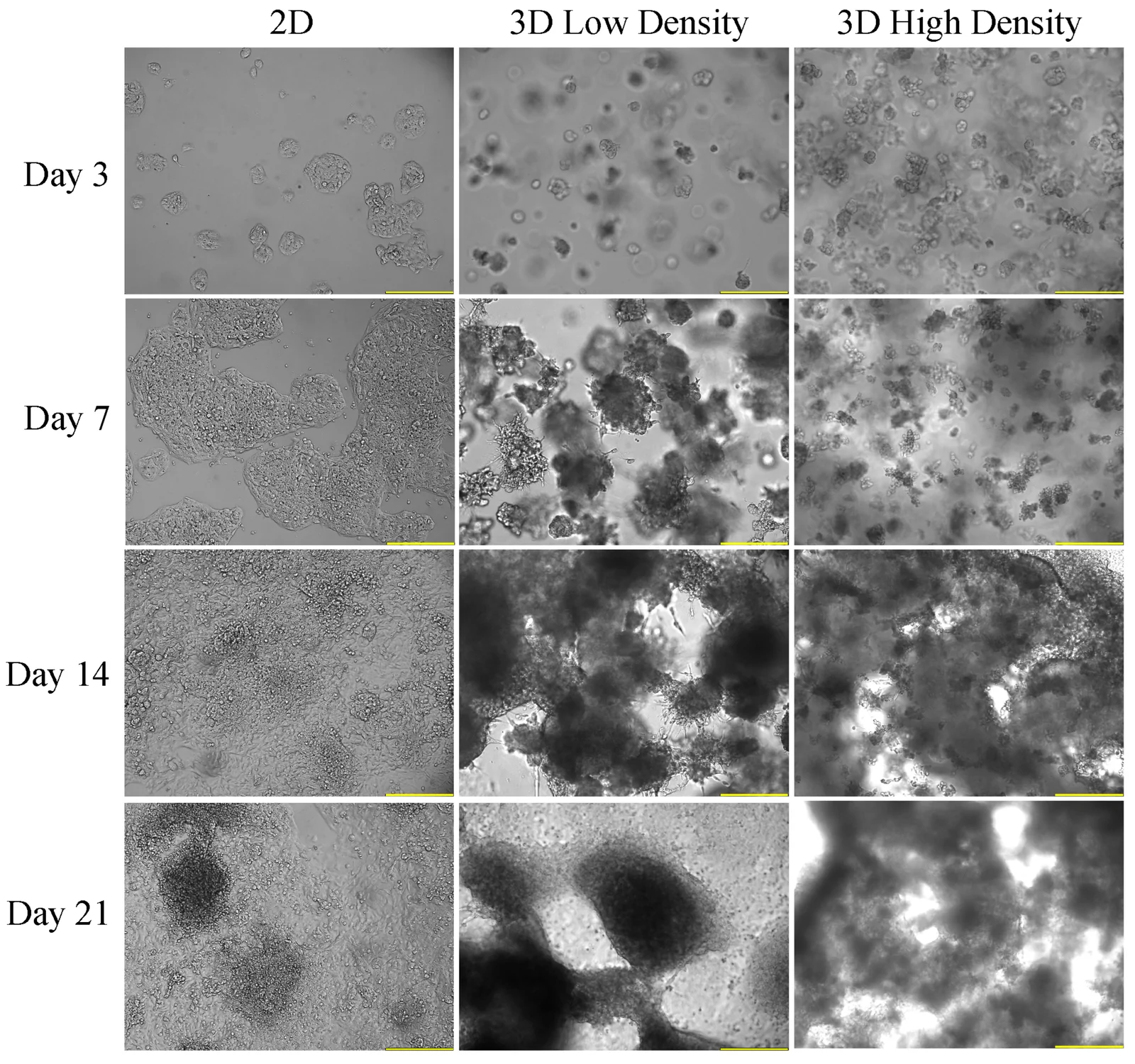
Establishment of C3A cell models. 2D, two-dimensional cultured cell model; 3D, three-dimensional cultured cell model; Scale Bar = 249 µm.

On the 3rd day after seeding, the 3D C3A model began to grow in spheroids with a small size. On the 7th day, the spheroid size in the low-density group increased significantly compared with that on the 3rd day, and tubular structures appeared at the edge of the spheroids, while the spheroids in the high-density group started to disintegrate. On the 14th and 21st days, some spheroids sedimented to the bottom of the plate and grew adherently, and the spheroids in the low-density group aggregated into larger-sized spheroids with dark black centers, the spheroids in the high-density group disintegrated more completely with poor cell status (Figure 1). Therefore, we concluded that the low-density 3D C3A model cultured for 7 days was a mature liver model, which was suitable for subsequent experiments.

### 3.2. Gene Expression of Cytochrome P450s

**Figure 2:**
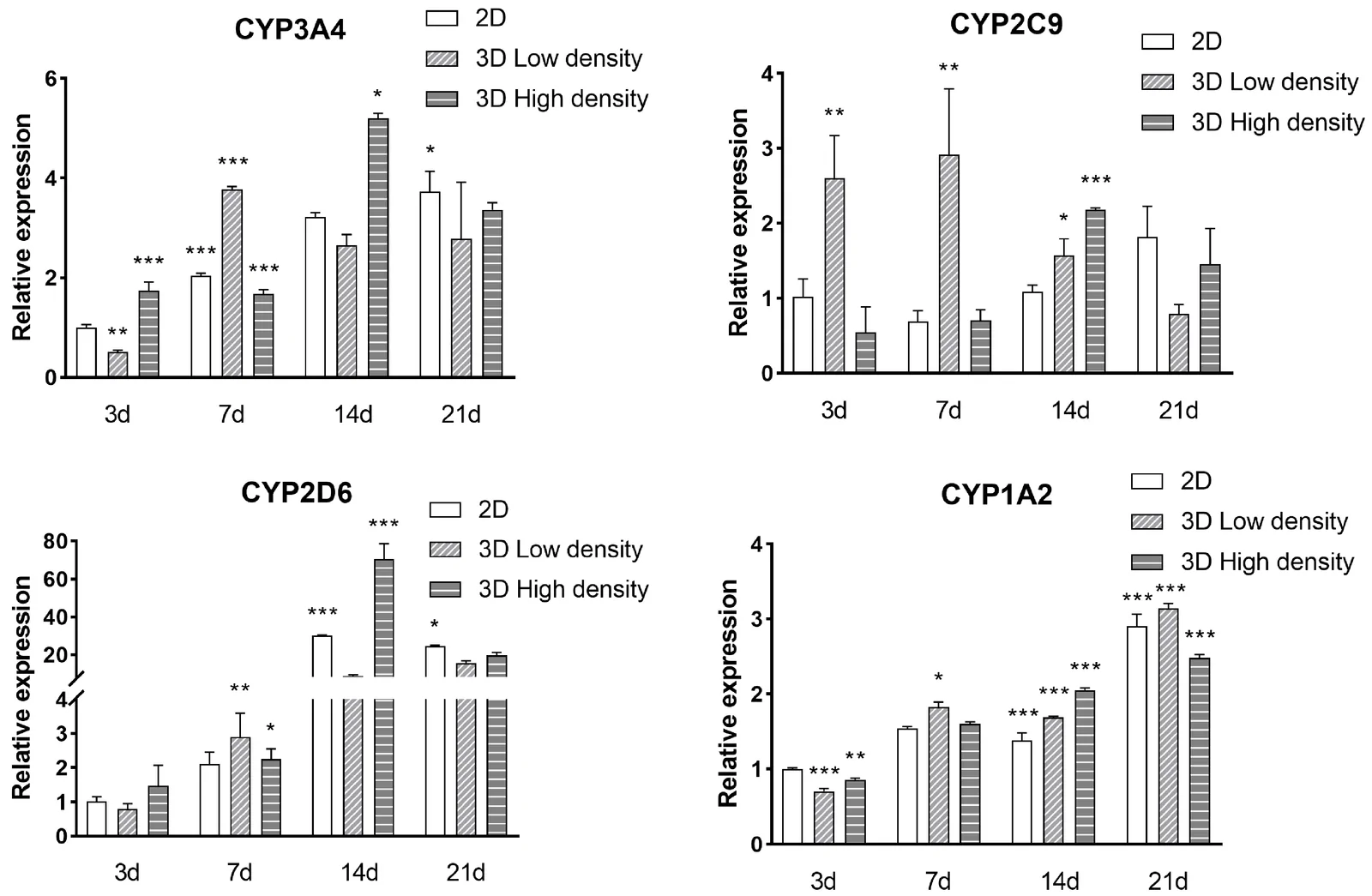
Gene expression of CYP3A4, CYP2C9, CYP2D6 and CYP1A2 in 2D and 3D C3A models. *: *p* < 0.05, **: *p* < 0.01, *** *p* < 0.001. The rest groups have no statistically significant difference.

The CYPs play a crucial role in drug metabolism, and their expression level is directly related to the applicability of the model in studies on drug metabolism and toxicity. RT-qPCR was used to detect the gene expression of CYP3A4, CYP2C9, CYP2D6 and CYP1A2 in 2D and 3D C3A models on the 3rd, 7th, 14th and 21st day after seeding. As shown in Figure 2, the gene expression of the CYPs in the low-density 3D C3A model on the 7th day was at a relatively high level, which was higher than that in the high-density model and the 2D cell model, and was also higher than that in the low-density 3D C3A model on the 3rd day. Although the expression of CYP2D6 and CYP1A2 showed an increasing trend after the 7th day, combined with the observations that spheroids in the low-density group aggregated into larger-sized spheroids with dark black centers, and spheroids in the high-density group underwent more complete disintegration accompanied by poor cell status in the subsequent days, and considerations for controlling the experimental cycle, the low-density 3D C3A model cultured for 7 days was selected for subsequent experiments.

### 3.3. Expression of Biomarkers

**Figure 3:**
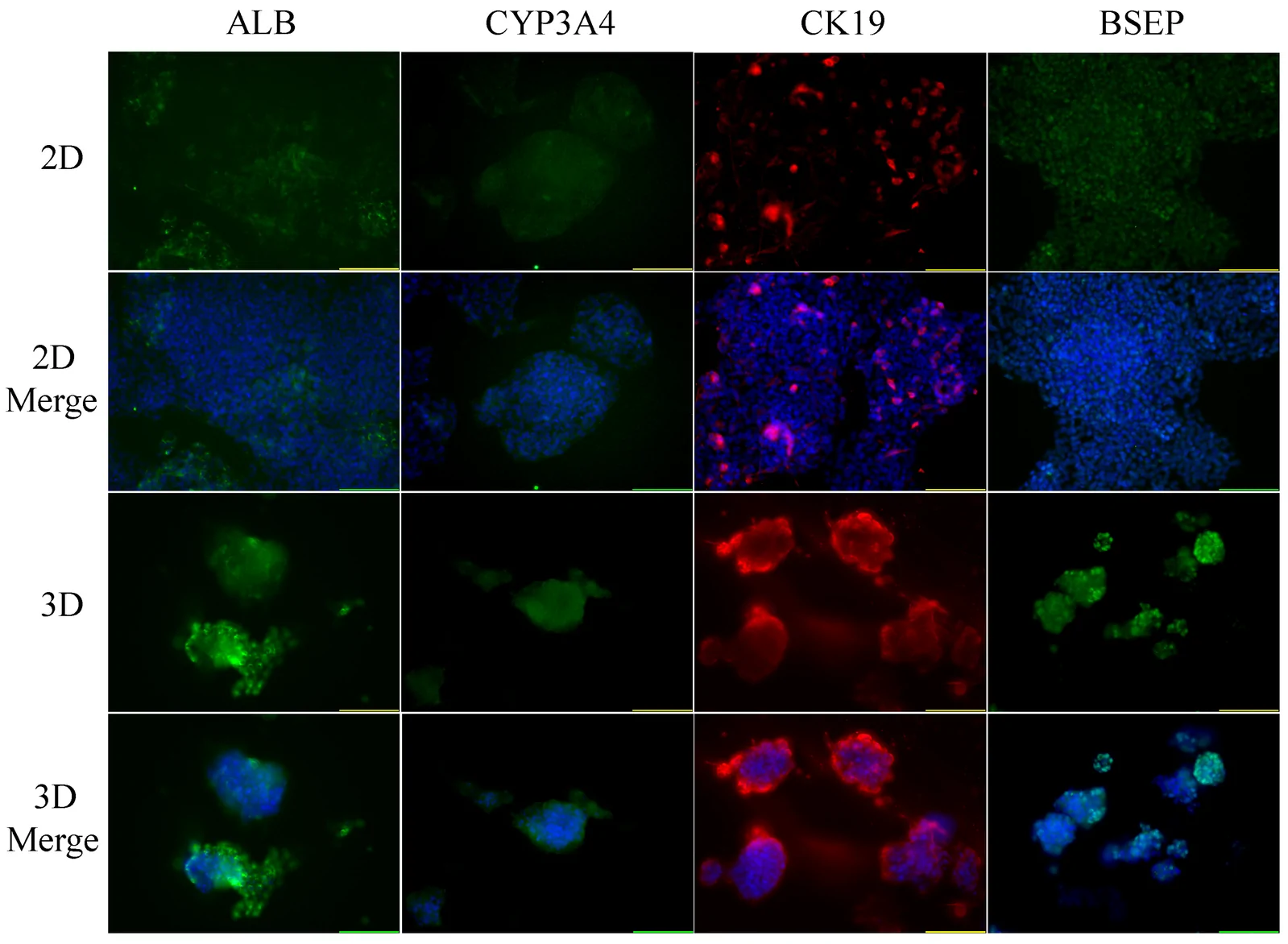
Expression of hepatobiliary-associated biomarkers in 2D and 3D C3A models. ALB, albumin; CYP3A4, cytochrome P450 3A4; CK19, cytokeratin 19; BSEP, bile salt export pump; Scale Bar = 249 µm.

Based on the result that the low-density 3D C3A model cultured for 7 days was suitable for subsequent experiments, immunofluorescence assays were performed to detect the expression of two liver biomarkers, ALB and CYP3A4, and bile duct biomarkers CK19 and BSEP in the low-density 3D C3A model cultured for 7 days, so as to further illustrate the potential of this model in DILI prediction. As shown in Figure 3, ALB was highly expressed in 2D C3A cells, and its expression was further enhanced in the 3D model. CYP3A4 was weakly expressed in 2D C3A cells, and its expression was upregulated in the 3D model. Surprisingly, the bile duct biomarkers CK19 and BSEP were both strongly expressed in both 2D and 3D C3A cell models. Further observation revealed the presence of partial CK19-positive tubular structures at the edge of 3D C3A spheroids, which was consistent with the observations that tubular structures appeared at the edge of the spheroids. Therefore, we proposed that the 3D C3A model exhibited more prominent hepatic and biliary duct characteristics compared with the 2D C3A cell model, making it suitable for DILI research and promising to improve the accuracy of DILI prediction.

### 3.4. Liver Toxicity Prediction Results

**Figure 4:**
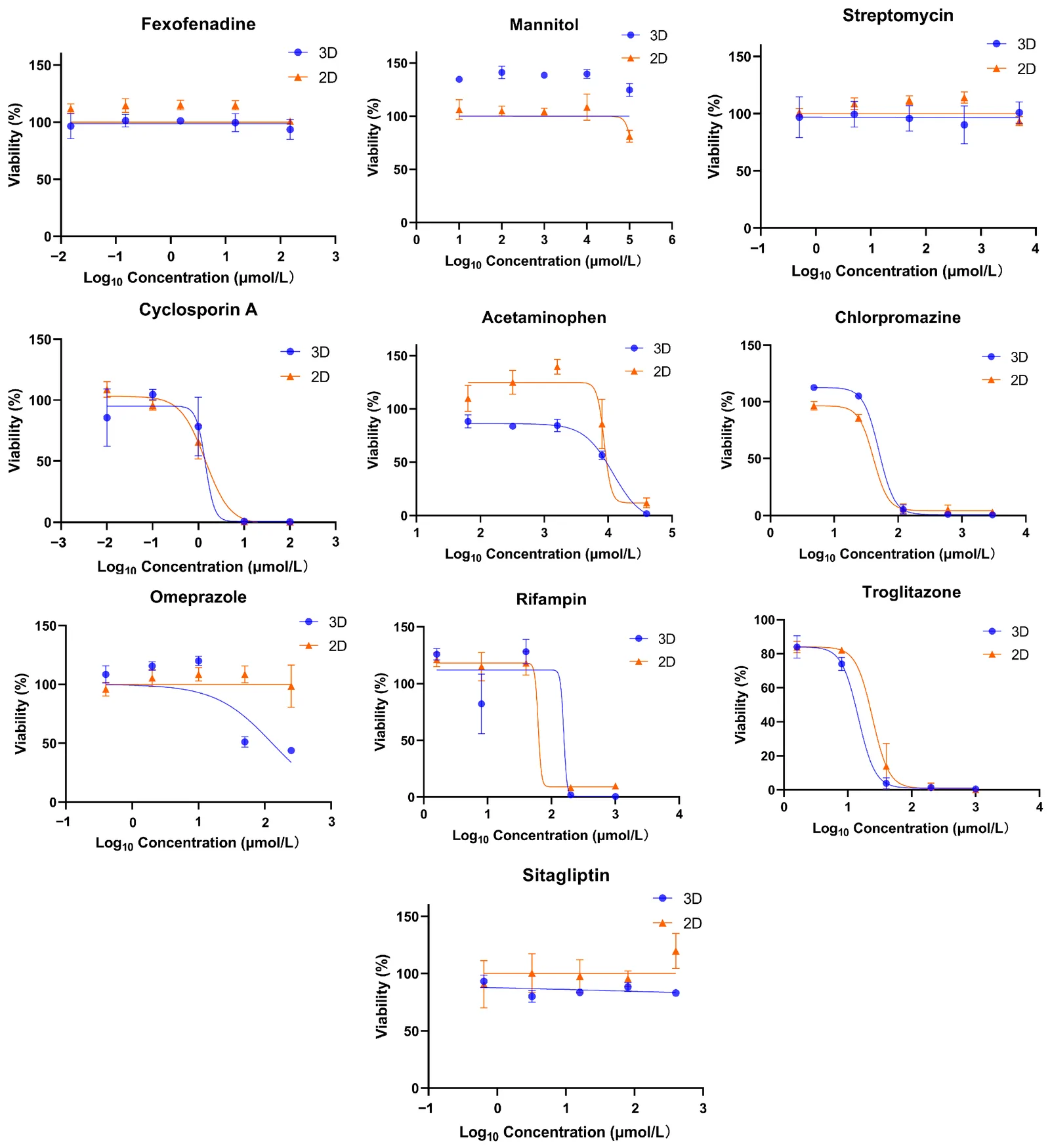
Results of DILI prediction. Drug half-maximal inhibitory concentration (IC50) was analyzed using GraphPad Prism 8.

Hepatotoxicity was determined using the ratio of IC50 to Cmax, with IC50/Cmax ≤ 50 classified as hepatotoxic and IC50/Cmax > 50 as non-hepatotoxic [32]. As shown in Table 3, fexofenadine, streptomycin, omeprazole, acetaminophen and sitagliptin were classified as non-hepatotoxic; mannitol, troglitazone, chlorpromazine, rifampin, and cyclosporin A were classified as hepatotoxic by using 2D C3A cell model for DILI prediction; while mannitol, fexofenadine, streptomycin, acetaminophen and sitagliptin were classified as non-hepatotoxic, troglitazone, omeprazole, chlorpromazine, rifampin, and cyclosporin A were classified as hepatotoxic by using 3D C3A cell model for DILI prediction. Based on clinical data, the sensitivity, specificity and accuracy of the 3D C3A model for DILI prediction were calculated using the formulas: Sensitivity = True positive/(True positive + False negative) × 100%, Specificity = True negative/(True negative + False positive) × 100%, and Accuracy = (True positive + True negative)/(True positive + False negative + True negative + False positive) × 100%. As shown in Figure 4 and Table 3, the sensitivity, specificity and accuracy of the 3D C3A model reached 83.3%, 100% and 88.9%, respectively, while those of the 2D C3A cell model were 66.7%, 66.7% and 66.7%, respectively. These results indicated that the 3D C3A model could predict DILI with better performance. In addition, neither the 2D nor the 3D model detected liver toxicity of sitagliptin.

### 3.5. Bile Duct Toxicity Prediction Results

**Figure 5:**
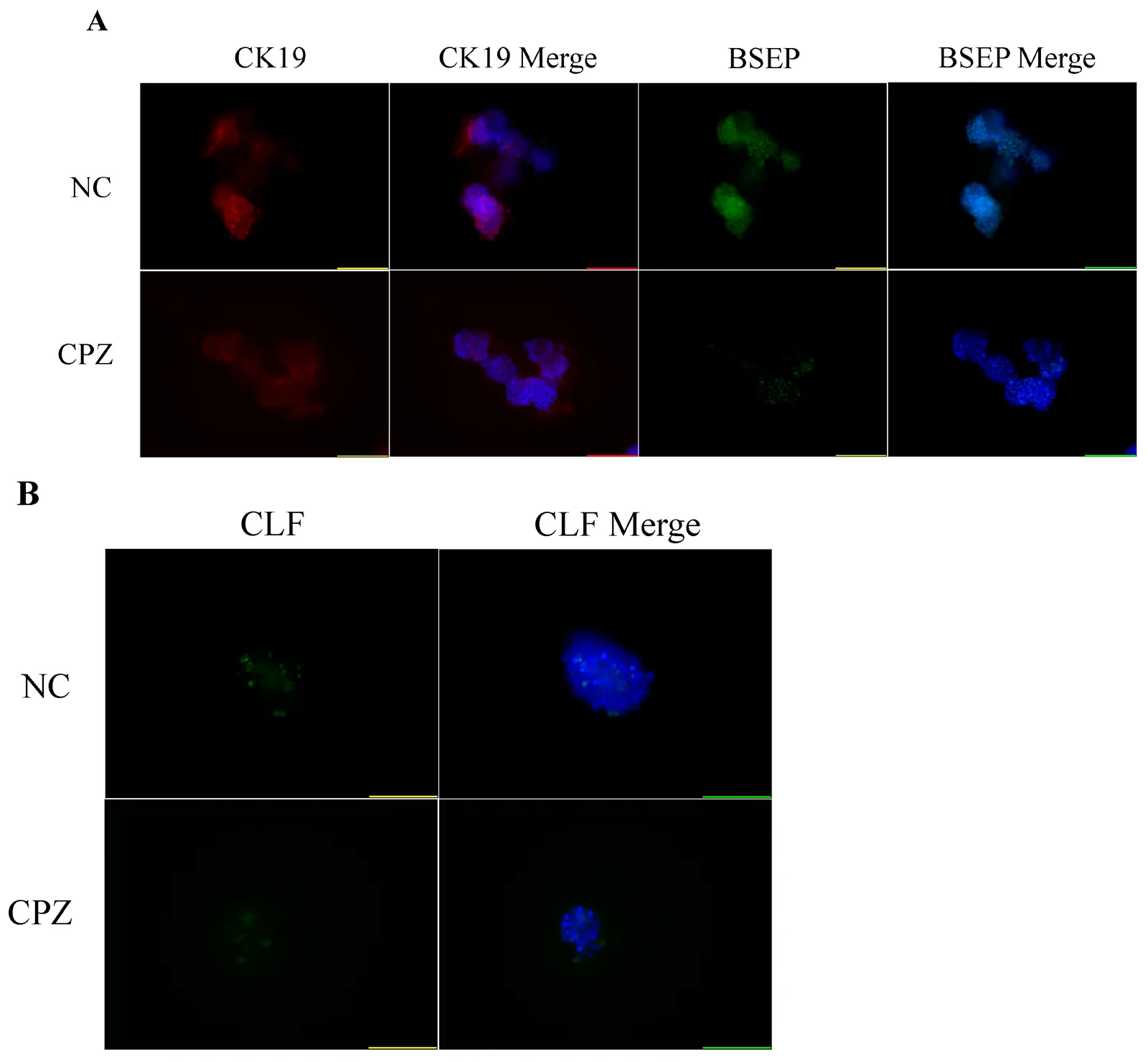
Results of drug-induced bile duct toxicity prediction. (**A**) Expression of bile duct biomarkers CK19 and BSEP was inhibited by CPZ. (**B**) Bile analog transport capacity was decreased after CPZ treatment. CPZ, chlorpromazine. Scale Bar = 249 µm.

Since the expression of biliary duct biomarkers CK19 and BSEP was detected in the 3D C3A model, we hypothesized that this model might also be applicable for predicting drug-induced biliary duct toxicity. Chlorpromazine, a drug with confirmed biliary duct toxicity, was selected for the experiment. Based on the results of the drug-induced liver toxicity test, the maximum non-hepatotoxic concentration (i.e., 24 μmol/L) was used in this experiment. As shown in Figure 5A, the expression of the biliary duct biomarkers CK19 and BSEP was downregulated in the model after chlorpromazine treatment, suggesting that chlorpromazine has potential biliary duct toxicity. The results of the bile analog (Cholyl–Lys–Fluorescein, CLF) transport assay showed that the bile analog transport capacity of the model was decreased after chlorpromazine treatment (Figure 5B), which further verified the biliary duct toxicity of chlorpromazine and also demonstrated that the model could be used to predict drug-induced biliary duct toxicity.

## 4. Discussion

Currently, NAMs are advancing rapidly, encompassing in vitro, in chemico, and in silico methodologies that are integrated to enhance human-relevant predictive accuracy while reducing animal reliance [33]. Three-dimensional liver models represent one category of in vitro technologies within this framework [34]. Researchers have constructed 3D liver models for predicting DILI. For example, 3D liver microtissues have been built using PHHs and non-parenchymal cells. Hepatotoxicity assessment of 110 compounds using these microtissues showed that 3D liver microtissues could identify hepatotoxic substances with greater sensitivity than 2D primary hepatocyte cultures [35]. Other researchers have generated liver organoids by using iPSCs [36,37]. These organoids exhibit albumin secretion levels comparable to PHHs, show upregulated expression of drug-metabolizing enzymes and transporter genes. This could enhance the sensitivity and specificity of DILI prediction and makes personalized DILI screening possible [36,37]. For example, Shinozawa et al. established human liver organoids derived from iPSCs, and functionally validated 238 marketed drugs, including 32 negative-control compounds and 206 reported DILI-associated compounds. Consequently, the model achieved a sensitivity of 88.7% and a specificity of 88.9% [38]. Furthermore, researchers have integrated biotechnology, tissue engineering, and microfluidics to successfully construct liver-on-a-chip devices. These chips express functional markers like ALB and drug-metabolizing enzymes. Microfluidic technology makes the chip closer to the real human liver environment. Therefore, liver chips show promising applications in predicting drug clearance rates and assessing DILI [39]. For instance, Emulate Inc. developed a liver-on-chip system incorporating PHHs, human liver sinusoidal endothelial cells, Kupffer cells, and stellate cells, and applied this platform to DILI prediction across 27 drugs, achieving 87% sensitivity and 100% specificity [32]. While the aforementioned methods can effectively improve the accuracy of DILI, they also have certain limitations. Using PHHs faces challenges in cell source availability, loss of function, and potential ethical issues. Using iPSC-derived organoids involves high economic costs and long construction periods. Using liver-on-a-chip technology requires highly skilled personnel and microfluidic devices. In addition, the lack of standardized construction protocols and validation criteria for liver organoids and liver-on-a-chip may lead to large inter-batch variability, and high cost and complicated operation also restrict their broad-scale application in routine drug safety screening.

This study focused on developing a simpler and more economical model, and more readily scalable model amenable to standardization, with comparisons against PHHs and other advanced 3D liver models, while maintaining predictive accuracy for DILI. Due to the advantages of the C3A cell line, such as easy accessibility, straightforward culture, low maintenance cost, and distinct hepatic characteristics, we selected the C3A cell line for model construction. The C3A cell line is a clonal derivative of HepG2, originally established from a hepatocellular carcinoma (HCC) specimen obtained from a 15-year-old patient [40]. A subset of HCCs co-expresses both hepatocyte markers and cholangiocyte markers, including CK19 [41,42]. During the experiments, we observed cholangiocyte-like features in C3A cells, including positive protein expression of CK19 and BSEP, and we attribute the appearance of CK19 in C3A cells to the inherent dual-phenotype nature of the parental tumor. These features also endowed the C3A model with potential for drug-induced bile duct injury prediction. Nevertheless, similar to HepG2, C3A cells exhibited weak CYP3A4 protein expression. We therefore sought to enhance expressions of CYPs through 3D culture. We explored optimal seeding density, culture duration, and treatment timing to enhance model maturity and the accuracy of DILI prediction. Based on the results shown in Figure 1 and Figure 2, we chose the 7-day low-density model for DILI prediction experiments. Extending the culture time further led to model disintegration and spheroid settling followed by adherent growth. Additionally, the 7-day low-density model showed advantages in the gene expressions of major CYPs, CYP3A4 and CYP2C9, key enzymes responsible for phase I drug metabolism in the liver. If the test compound is primarily metabolized by CYP2D6 or CYP1A2, lowering the cell seeding density and extending the model maturation time to 14 or 21 days could be considered. This would allow the expression levels of the corresponding metabolic enzymes to reach higher levels, better simulating in vivo drug metabolism for improved DILI prediction and research.

Based on FDA-published data, the clinical data, and relevant literature [19,20,21,22,23,24,25,26,27,28,29], we selected three drugs with no hepatotoxicity, six drugs with hepatotoxicity, and one drug with ambiguous hepatotoxicity for prediction studies. The results demonstrated that the 3D C3A cell model achieved a sensitivity of 83.3% and a specificity of 100%, suggesting potential advantages over 2D PHHs, for which reported results include 40.6% sensitivity and 85.4% specificity [5], and 26% sensitivity and 100% specificity [43]. Li et al. assessed hepatotoxicity for a panel of 100 compounds using 2D and 3D PHHs, with hepatotoxicity classified according to criteria identical to those applied in the present study [43]. Among these compounds, streptomycin, acetaminophen, troglitazone, chlorpromazine and rifampin overlapped with compounds tested in our work. Notably, for non-hepatotoxic streptomycin, 2D PHHs gave a correct non-hepatotoxic prediction, but 3D PHHs generated a false-positive hepatotoxic result, whereas our 3D C3A model correctly classified streptomycin as non-hepatotoxic. For rifampin (a known hepatotoxic compound), both 2D and 3D PHHs failed to capture its hepatotoxic risk, in contrast to the correct prediction obtained with our model. As for troglitazone and chlorpromazine, which are hepatotoxic, 2D and 3D PHHs accurately predicted their hepatotoxicity, in agreement with our 3D C3A model results. For the hepatotoxic drug acetaminophen, 2D PHHs failed to predict its hepatotoxicity, whereas 3D PHHs correctly classified it as hepatotoxic. However, both our 2D and 3D C3A models produced false-negative outcomes for acetaminophen. A plausible explanation is that C3A cells exhibit low expression of the CYPs required for acetaminophen metabolism, resulting in insufficient production of toxic metabolites [44]. In PHHs, 2D culture led to rapid loss of activities of CYPs, whereas 3D culture sustained CYPs function for longer periods, accounting for the divergent predictive performance between 2D and 3D PHHs toward acetaminophen. Another study evaluated acetaminophen-induced hepatotoxicity in 2D PHHs using alanine aminotransferase (ALT) activity assays and nuclear propidium iodide (PI) staining for necrosis, and detected hepatotoxicity following 48 h exposure to 10 mmol/L acetaminophen [45]. This finding indicated that even 2D PHHs were capable of capturing acetaminophen-induced toxicity under appropriate assay conditions. Therefore, elevating CYP expression in C3A cells and combining multiple endpoints such as biochemical markers (ALT, ALB) and PI staining may help improve DILI-prediction performance. Both our 2D and 3D C3A models correctly predicted the hepatotoxicity of cyclosporin A, which was consistent with the findings reported by Zhong et al. using 3D-cultured PHHs [46]. And the non-hepatotoxic drug mannitol exhibited no hepatotoxicity in both our 3D C3A model and the PHH-based Liver Chip model [47] but showed hepatotoxicity in 2D C3A model. This might be because, in the 2D culture, all cells were directly exposed to mannitol, increasing the contact area between cells and the drug and leading to abnormal toxicity. In contrast, the 3D model, with its spheroidal growth, was physiologically closer to human liver tissue, and the cell–drug contact area more closely mimicked the in vivo situation, thus no hepatotoxicity was detected. The hepatotoxic drug omeprazole was correctly identified as toxic in the 3D model but not in the 2D model. This could be because the expressions of the metabolizing enzymes CYP2C19 and CYP3A4 were higher in the 3D model than in the 2D model [48]. After 3 days of omeprazole incubation, more toxic metabolites were generated in the 3D model, whereas the weaker CYP enzyme expression in the 2D model failed to produce sufficient toxic metabolites. Sitagliptin, a DPP-4 inhibitor used for type 2 diabetes, is primarily excreted by renal, with minor hepatic metabolism. Clinical trials involving 14,611 patients found no hepatotoxicity [49]. However, there is a single case report documenting liver injury in two patients after sitagliptin use [29]. Consequently, the DILIrank database classifies sitagliptin as Less-DILI-Concern [50]. Considering our experimental results, we consider that the rare hepatotoxicity associated with sitagliptin might be related to individual variations, such as abnormal expression or mutations in the patient’s liver metabolic enzymes.

It is important to note that this study used IC50/Cmax ≤ 50 as the criterion for hepatotoxicity [32], which was suitable for our experimental system. However, experimental systems established under different conditions may require adjustments to this criterion based on the specific context. Factors such as the experimental environment, model construction method, and cell source can influence the final results. Therefore, establishing appropriate criteria requires sufficient experimental exploration based on data from a wide range of drugs and corresponding clinical data. Additionally, this study did not validate the hepatotoxicity of large molecule drugs; if the model is to be used for testing such drugs, we recommend removing the Matrigel and collecting the spheroids for toxicity testing. This is because Matrigel might adsorb some large molecules or physically hinder their penetration to the spheroids, potentially leading to false-negative results.

An unexpected and novel finding in this study was that the established 3D C3A model exhibited both hepatic properties and characteristics of the bile duct. Robust expression of bile duct markers, especially the presence of labeled tubular structure at the periphery of spheroids within this 3D model, is rarely observed in other hepatic models. This led us to hypothesize that the model might not only be useful for predicting DILI but also for predicting drug-induced bile duct injury. Therefore, we conducted experiments using chlorpromazine, a well-characterized cholestatic toxicant known to induce bile duct injury. The maximum non-hepatotoxic concentration was selected to minimize the confounding effect of drug hepatotoxicity on the assessment of bile duct toxicity. The results showed that the maximum non-hepatotoxic concentration of chlorpromazine reduced the expression of the bile duct marker CK19 and BSEP. Decreased expression of BSEP would lead to the accumulation of bile salts within hepatocytes due to impaired transport function, which eventually resulted in hepatic injury. This was further supported by CLF transport assays. Therefore, we believe the 3D C3A model can, to some extent, predict both DILI- and drug-induced bile duct injury. Furthermore, it holds potential for exploring the molecular mechanisms behind liver injury triggered by bile duct damage and cholestasis.

Undeniably, PHHs remain the most widely accepted 2D cell model in the field of DILI prediction. They are widely used in constructing in vitro multicellular liver models and liver-on-a-chip systems and play a crucial role in drug hepatotoxicity research [51]. However, several inherent characteristics of PHHs constrain their application. Donor variability leads to divergent drug responses, compromising experimental consistency and reproducibility; limited availability results in low utilization efficiency and elevated costs for DILI prediction, and functional decline in culture; specifically, rapid functional decay characterized by loss of albumin secretion and activities of CYPs in 2D monolayers causes insufficient drug metabolism, thereby affecting DILI predictive accuracy [52,53]. iPSCs offer unlimited expansion potential; hepatocytes derived from iPSCs could overcome the source limitation of PHHs. Therefore, establishing stable differentiation protocols based on iPSCs and optimizing these systems to shorten the time frame and reduce costs is crucial [54]. Furthermore, the mechanisms by which different drugs induce liver injury are not uniform [55]. Some drugs induce injury through toxic metabolites generated by metabolic enzymes, others through oxidative stress, some by inhibiting bile acid transporters leading to cholestasis, and others by disrupting intrahepatic vasculature. Thus, constructing different types of functional 3D liver models or liver-on-a-chip models could provide reliable tools for DILI prediction and related mechanistic studies. We will continue our efforts to develop more comprehensive models with broader functionality, lower cost, and enhanced stability.

## 5. Conclusions

In this study, a 3D liver model, which was simple, low-cost, easy to culture, and more readily scalable model, in comparison with conventional 2D PHHs and other advanced 3D liver models, was constructed and applied in DILI prediction. The entire prediction procedure took only 10 days, and the sensitivity, specificity and accuracy of the model reached 83.3%, 100% and 88.9%, respectively. Notably, this model was potentially applicable not merely to the prediction of DILI, but also to drug-induced bile duct injury prediction. Nevertheless, this study still had room for improvement. This study evaluated only 10 test compounds. The limited size of the compound dataset may restrict the robustness and generalizability of the 3D C3A model for broad-spectrum prediction of DILI. Further validation with a larger panel of diverse hepatotoxic and non-hepatotoxic compounds should be performed in future studies. In addition, this model remains to be further improved; future improvements may focus on elevating CYPs expression levels in C3A cells, and additional detection assays including ALB quantification and PI staining could be adopted to achieve comprehensive evaluation of drug-induced hepatotoxicity. Moreover, integrating Kupffer cells, hepatic stellate cells and liver sinusoidal endothelial cells to construct a multicellular liver model would enhance its simulation performance. Despite these shortcomings, our 3D liver model holds considerable application potential in preclinical drug screening, hepatotoxicity assessment, and translational research. It could facilitate high-throughput preliminary toxicity screening at the early stages of drug development, aid in lead-compound prioritization, and mitigate over-reliance on animal studies for initial toxicological evaluations.

## Figures and Tables

**Table 1:** Primers for RT-qPCR.

Name	Sequence (5′ → 3′, Forward)	Sequence (5′ → 3′, Reverse)
CYP3A4	AGAACTCTGAAATGAAGATGGGC	TCCCGTGAGAAGCAGAGGAGCCAA
CYP2D6	GCATAGTGGTGGCTGACCTGTTCT	ATCACGTCGTCGATCTCCTGTTGG
CYP2C9	CCTCTGGGGCATTATCCATC	ATATTTGCACAGTGAAACATAGGA
CYP1A2	GCCATTACAACCCTGCCAATCTCA	CAGTGCCAGGTGCGGGTTCTTC
GAPDH	CTCTGCTCCTCCTGTTCGAC	TTAAAAGCAGCCCTGGTGAC

**Table 2:** Concentration of Drugs.

Drug	Concentration (μmol/L)
Mannitol	10, 100, 1000, 10,000, 100,000
Fexofenadine	0.015, 0.15, 1.5, 15, 150
Streptomycin	0.5, 5, 50, 500, 5000
Troglitazone	1.6, 8, 40, 200, 1000
Omeprazole	0.4, 2, 10, 50, 250
Chlorpromazine	4.8, 24, 120, 600, 3000
Rifampin	1.6, 8, 40, 200, 1000
Cyclosporin A	0.01, 0.1, 1, 10, 100
Acetaminophen	64, 320, 1600, 8000, 40,000
Sitagliptin	0.64, 3.2, 16, 80, 400

**Table 3:** Results of DILI prediction.

Drug	Classification	Culture Dimension	IC50	Cmax	IC50/Cmax	Result
Mannitol	Non-hepatotoxic	2D	130.27 mmol/L	7.46 mmol/L	17.46	Hepatotoxic
		3D	ND		ND	Non-hepatotoxic
Fexofenadine	Non-hepatotoxic	2D	ND	1.40 μmol/L	ND	Non-hepatotoxic
		3D	ND		ND	Non-hepatotoxic
Streptomycin	Non-hepatotoxic	2D	ND	52.27 μmol/L	ND	Non-hepatotoxic
		3D	640.60 mmol/L		1.23 × 10^4^	Non-hepatotoxic
Troglitazone	Hepatotoxic	2D	24.24 μmol/L	6.39 μmol/L	3.79	Hepatotoxic
		3D	14.57 μmol/L		2.28	Hepatotoxic
Omeprazole	Hepatotoxic	2D	ND	2.93 μmol/L	ND	Non-hepatotoxic
		3D	129.50 μmol/L		44.20	Hepatotoxic
Chlorpromazine	Hepatotoxic	2D	40.95 μmol/L	3.47 μmol/L	11.80	Hepatotoxic
		3D	49.97 μmol/L		14.40	Hepatotoxic
Rifampin	Hepatotoxic	2D	61.79 μmol/L	18.96 μmol/L	3.26	Hepatotoxic
		3D	155.40 μmol/L		8.20	Hepatotoxic
Cyclosporin A	Hepatotoxic	2D	1.41 μmol/L	0.68 μmol/L	2.07	Hepatotoxic
		3D	1.42 μmol/L		2.09	Hepatotoxic
Acetaminophen	Hepatotoxic	2D	8.67 mmol/L	147.60 μmol/L	58.74	Non-hepatotoxic
		3D	11.77 mmol/L		79.74	Non-hepatotoxic
Sitagliptin	Ambiguous	2D	ND	3.95 μmol/L	ND	Non-hepatotoxic
		3D	1.19 × 10^15^ μmol/L		3.01 × 10^14^	Non-hepatotoxic

ND, Not Detected.

## Data Availability

The original contributions presented in the study are included in the article. Further inquiries can be directed to the corresponding authors.

## Abbreviations

The following abbreviations are used in this manuscript:

DILI	Drug-induced liver injury
ECM	Extracellular matrix
2D	Two-dimensional
3D	Three-dimensional
IC50	Half-maximal inhibitory concentration
Cmax	Maximum plasma concentration
CPZ	Chlorpromazine
PHHs	Primary human hepatocytes
CK19	Cytokeratin 19
BSEP	Bile salt export pump
ALB	Albumin
CYPs	Cytochrome P450 enzymes
AFP	Alpha-fetoprotein
iPSCs	induced pluripotent stem cells
HCC	Hepatocellular carcinoma
ALT	Alanine aminotransferase
PI	Propidium iodide
CLF	Cholyl-Lys-Fluorescein
